# Combining clinical and imaging data for predicting functional outcomes after acute ischemic stroke: an automated machine learning approach

**DOI:** 10.1038/s41598-023-44201-8

**Published:** 2023-10-07

**Authors:** Hongju Jo, Changi Kim, Dowan Gwon, Jaeho Lee, Joonwon Lee, Kang Min Park, Seongho Park

**Affiliations:** 1 Department of CGMS Sensor, Sensor R&D Center, i-SENS, Seoul, Republic of Korea; 2 Integrated Major in Innovative Medical Science, Seoul National University Graduate School, Seoul, Republic of Korea; 3 Department of Digital&Biohealth, Group of AI/DX Business, KT, Seoul, Republic of Korea; 4 Department of Preventive Medicine, Seoul National University College of Medicine, Seoul, Republic of Korea; 5 Department of Neurology, Haeundae Paik Hospital, Inje University College of Medicine, Haeundae-ro 875, Haeundae-gu, 48108 Busan, Republic of Korea

**Keywords:** Neurology, Risk factors

## Abstract

This study aimed to develop and validate an automated machine learning (ML) system that predicts 3-month functional outcomes in acute ischemic stroke (AIS) patients by combining clinical and neuroimaging features. Functional outcomes were categorized as unfavorable (modified Rankin Scale ≥ 3) or not. A clinical model employing optimal clinical features (Model_A), a convolutional neural network model incorporating imaging data (Model_B), and an integrated model combining both imaging and clinical features (Model_C) were developed and tested to predict unfavorable outcomes. The developed models were compared with each other and with traditional risk-scoring models. The dataset comprised 4147 patients from a multicenter stroke registry, with 1268 (30.6%) experiencing unfavorable outcomes. Age, initial NIHSS, and early neurologic deterioration were identified as the most important clinical features. The ML model prediction achieved an area under the curves of 0.757 (95% CI 0.726–0.789) for Model_A, 0.725 (95% CI 0.693–0.755) for Model_B, and 0.786 (95% CI 0.757–0.814) for Model_C in the test set. The integrated models outperformed traditional risk-scoring models by 0.21 (95% CI 0.16–0.25) for HIAT and 0.15 (95% CI 0.11–0.19) for THRIVE. In conclusion, the integrated ML system enhanced stroke outcome prediction by combining imaging data and clinical features, outperforming traditional risk-scoring models.

## Introduction

Prognosis related to functional outcomes following a stroke is a major concern for patients and their families. Physicians need to be able to predict functional recovery when establishing long-term treatment plans^1^. Functional outcomes are influenced by whether the infarction occurred in a motor-related eloquent area^2,3^. Thus, utilizing imaging information about the location and extent of brain lesions is crucial for predicting patient prognosis^4,5^.

Convolutional neural networks (CNNs), a type of deep neural network, can effectively process spatial information from lesions. CNN models using stroke images are anticipated to provide better prognostic predictions after acute stroke. However, most traditional models for predicting stroke functional prognosis to date have relied on risk score systems that use only non-imaging clinical features or imaging-derived features^6–9^. Recently, several machine learning (ML) based prediction models have been proposed, but the majority did not incorporate lesion imaging information^10^.

A multi-modal system that combines multiple types of data, instead of using each data type alone, has been proposed to improve the model's performance by increasing the amount of information^11–13^. Nevertheless, few multimodal systems have been developed to predict stroke outcomes^10,14^.

The limited explainability and usability of existing ML models have hindered their practical application. Deep learning algorithms, such as CNNs, are often considered "black box" models, making them difficult to interpret. ML systems that require clinicians to input extensive information and preprocess data tend to be less user-friendly.

There is still a need for reliable ML-based prediction models that can be rapidly and easily applied in real-world practice. Therefore, considering the aforementioned limitations, we aimed to develop an automated ML system that predicts 3-month functional outcomes by combining a CNN model using imaging data with a clinical model utilizing a few optimal clinical features. We conducted a validation of the developed model, comparing its performance to that of traditional prediction models. Additionally, we sought to provide better explanations using a model-specific interpretation method called class activation mapping^15^.

## Methods

### Study design and data sources

This diagnostic test accuracy study employed a diagnostic cohort design, aiming to develop and evaluate a machine learning (ML) system for predicting functional outcomes three months after stroke. The dataset was acquired from the National Information Society Agency (NIA), which was prepared for developing an artificial intelligence model based on a multi-center prospective stroke registry. The dataset contains imaging and clinical data from acute ischemic stroke patients registered between January 2011 and March 2019 at three university hospitals' stroke centers in the Republic of Korea. The imaging data included diffusion-weighted images (DWI, b = 1000 s/mm^2^), apparent diffusion coefficient (ADC) images, and manually segmented infarction lesion labels. From the original dataset of 6000 patients, 600 with unavailable clinical data were excluded. Among the remaining 5400 eligible patients, those under 18 years old, with a pre-stroke modified Rankin scale (mRS)^16^ score of three or higher, and without follow-up mRS evaluation were excluded. Table S1 provides information on the 31 common clinical variables used in our study, selected from all variables in the NIA dataset.

All data were double-reviewed by experienced neurologists (P. K. M and L. J) who were blinded to the study design. This study adhered to the TRIPOD^17^ and CLAIM^18^ reporting guidelines. Data were anonymized using each data provider's de-identification method (Fig. S1). The study was approved by the Institutional Review Board (IRB No. 20180709) of Haeundae Paik Hospital, and the requirement for informed consent was waived due to the study's retrospective design.

### Definition of functional outcome

A functional outcome was defined as a binary label, with an unfavorable outcome if the mRS was three or higher and a favorable outcome if less than three^16^.

### Data preparation

Methods for clinical data preparation and feature selection are provided in Method S1. Method S2 contains MRI acquisition protocols for brain imaging data. All acquired DWIs, ADCs, and lesion masks underwent identical image pre-processing steps (Fig. S2).

### Model development

To classify three-month binary functional outcomes, we developed and tested prediction models for performance validation. We randomly allocated 20% of the dataset as a test set, exclusively for evaluation. The remaining 80% served as a training set for hyperparameter determination and training, employing fivefold cross-validation (Fig. S3).

We developed the ML model as follows. First, we constructed Model A, a clinical model, using ML algorithms such as logistic regression (LR), random forest (RF), light gradient boosting machine (LGBM)^19^, and multi-layer perceptron (MLP)^20^, based on selected variables from the clinical dataset. Second, we built Model B, an imaging model, by training a deep three-dimensional DenseNet (CNN model) on imaging data with three channels (DWI, ADC, and ground truth lesion mask)^21^. Third, we developed an integrated ML model (Model C) using prediction probabilities from Model B and selected variables from the clinical dataset as multi-modal inputs. Lastly, we also developed a lesion segmentation model (Model S) for the same imaging data, utilizing a two-dimensional U-Net model with a ResNet152 backbone^22^. Model S was designed to create a pipeline that automatically processes segmentation and is assigned to the model's input if the user inputs only the original DWI and ADC without performing manual segmentation. We evaluated Model B using the predicted mask from Model S instead of the ground truth mask.

All developed models were evaluated for performance using the test set (unseen dataset). The overall flow diagram of ML model development and evaluation is shown in Fig. S4.

### Performance evaluation

We used the area under the receiver operating characteristic curve (AUC) as the primary performance metric to evaluate the outcome prediction model. Initially, we assessed the performance of the LR, RF, LGBM, and MLP algorithms using the test set. We then adopted one algorithm as the representative ML model. With the representative ML model, we compared the performances of Models A, B, and C. Next, we plotted a calibration plot and calculated the Brier score, which represents the mean squared difference between the predicted probability and the true outcome, to evaluate the models' calibration performances^23,24^. Finally, we compared the performance of Model C on the test set with traditional models (HIAT and THRIVE)^6,7^. We used the intersection over union (IoU) and Dice similarity coefficient (DSC) as performance evaluation metrics for lesion area detection in the segmentation model (Model S)^25,26^.

### Visual explanations from deep networks

We employed Grad-CAM to visualize the saliency map for Model B, a CNN that classifies functional outcomes^15^. This method is useful for understanding which parts of the image contribute to the CNN's final classification decision. We examined several cases where the model's predicted outcome matched the actual outcome and those with discrepancies.

### Statistical analysis

Values are presented as mean ± standard deviation, median (interquartile range) for continuous variables, or as number (%) of subjects for categorical variables, as appropriate. We compared the clinical characteristics between two groups using the Chi-square test, Fisher's exact test, Mann–Whitney test, or Student's t-test, depending on the type of variable. To assess the models' performance in discriminating three-month functional outcomes, we plotted receiver operating characteristic (ROC) curves and calculated the area under the curve (AUC) and 95% confidence interval (CI) for each model. We compared differences between AUCs using DeLong's test^27^. Furthermore, we calculated positive predictive value, negative predictive value, and Brier scores as secondary outcome metrics. We also determined sensitivity and specificity values for the threshold defined by Youden's index J (J = sensitivity + specificity—1), if necessary. We conducted statistical analyses using MedCalc software (version 20.114) for generating the ROC curve and performing DeLong's test. We carried out other statistical analyses using R version 4.1.2. We considered a 2-tailed P-value < 0.05 to be statistically significant.

## Results

A total of 4147 patients were included in the dataset, with 1268 (30.6%) experiencing an unfavorable outcome (Fig. S5). All missing values for the common dataset variables were within 10% (Fig. S6), leading us to apply multivariate imputation-chained equations to all variables. The mean age was 68.09 ± 12.6 years, and 1765 (61.3%) were men. Baseline demographics and clinical characteristics based on favorable and unfavorable outcomes of the dataset, with imputation performed, are listed in Table S2.

We calculated feature importance and contribution to the outcome for the 31 imputed common variables (Fig. S7). Age, initial NIHSS, and early neurologic deterioration (END) were consistently identified as the most important variables in Random Forest feature importance, permutation importance, and SHAP value analyses. The direction of feature contribution shown in the SHAP summary plot was in line with general clinical interpretation (Fig. S8). For most of the remaining features, the relationship between the distribution of variable values and SHAP values was more heterogeneous. Considering the concern of overfitting and the advantage of selecting the minimum features to use as input variables in clinical practice, we ultimately chose age, initial NIHSS, and END as the ML prediction system's clinical input features.

**Figure 1:**
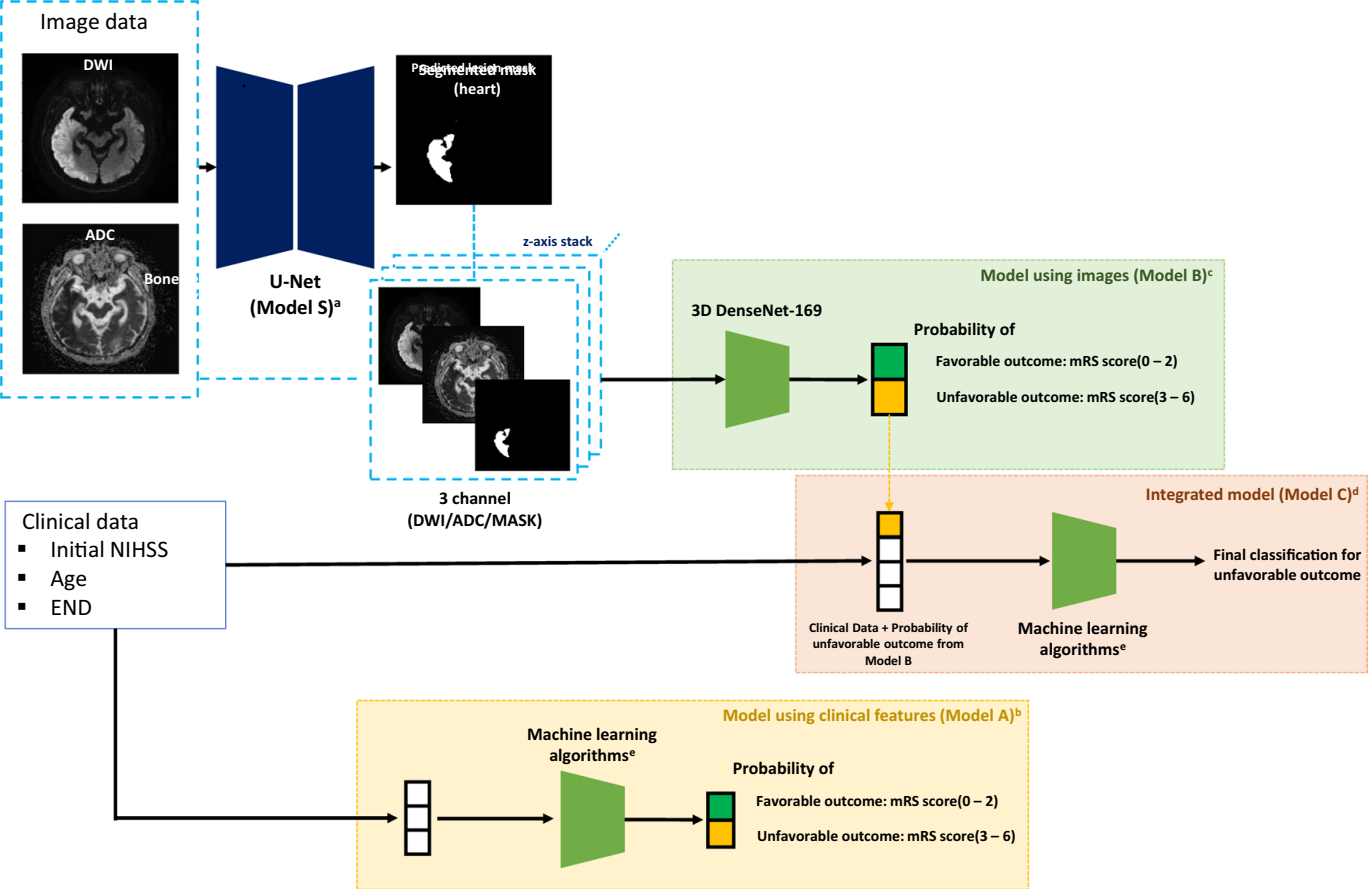
Pipeline of proposed prediction model for classifying 3-month functional outcome. *DWI* diffusion-weighted imaging, *ADC* apparent diffusion coefficient, *mRS* modified-Rankin Scale, *NIHSS* National Institutes of Health Stroke Scale, *END* early neurologic deterioration. ^a^Model S : Lesion segmentation model using U-Net with a ResNet152 backbone. ^b^Model A : Model using only clinical features (Initial NIHSS, age, END). ^c^Model B : Model using only images (DWI, ADC and predicted lesion mask). ^d^Model C: Model incorporating the probability estimates for unfavorable outcomes from Model B and integrates them with the clinical features (initial NIHSS, age, and END). ^e^Machine learning algorithms : Logistic regression, random forest, light gradient boosting model, multilayer perceptron.

Figure 1 illustrates the overall pipeline of class prediction for the three-month functional outcome using the developed models, with model development details provided in Tables S3–S5.

### Performance of the models

Model S achieved an overall mean Dice score of 0.8433 and an IoU of 0.7968. It selectively segmented only the high signal attributed to the infarcted core, excluding high signals that could be mistaken for diffusion restriction, such as susceptibility artifacts (Fig. S9).

Table 1 presents the performance estimates of the ML algorithms and CNN. Among the four ML algorithms, the performance was quite similar, but random forest (RF) demonstrated the highest performance. As a result, we chose RF as the representative ML algorithm for Models A and C.

**Table 1:** Comparison of the performance of models predicting 3-month functional outcomes on the test set (n = 822).

	Model A^a^				Model B^b^	Model C^c^			
	LR	RF	LGBM	MLP	CNN	LR	RF	LGBM	MLP
AUROC (95% CI)	0.764 (0.734–0.793)	0.757 (0.726–0.786)	0.755 (0.724–0.784)	0.764 (0.734–0.793)	0.725 (0.693–0.755)	0.783 (0.753–0.811)	0.786 (0.757–0.814)	0.787 (0.757–0.814)	0.786 (0.757–0.814)
Sensitivity (95% CI)	75.1 (69.2–80.4)	73.5 (67.5–78.9)	67.8 (61.5–73.6)	79.2 (73.6–84.1)	80.0 (74.4–84.8)	81.6 (76.2–86.3)	78.0 (72.2–83.0)	79.6 (74.0–84.5)	79.2(73.6–84.1)
Specificity (95% CI)	69.2 (65.2–72.9)	70.0 (66.1–73.7)	75.2 (71.5–78.7)	64.5 (60.4–68.4)	55.6 (51.5–59.7)	63.3 (59.2–67.2)	66.2 (62.2–70.1)	64.8 (60.8–68.7)	65.9 (61.8–69.7)
PPV (95% CI)	50.8 (47.3–54.4)	51.1 (47.4–54.6)	53.7 (49.6–57.8)	48.6 (45.5–51.8)	43.4 (40.7–46.1)	48.5 (45.5–51.6)	49.5 (46.2–52.8)	49 (45.8–52.2)	49.6 (46.4–52.9)
NPV (95% CI)	86.7 (83.9–89.1)	86.1 (83.4–88.5)	84.6 (82.0–86.9)	87.9 (85.0–90.4)	86.8 (83.5–89.5)	89.0 (86.1–91.4)	87.6 (84.7–90.0)	88.2 (85.3–90.6)	88.2 (85.3–90.5)

*AUROC* area under the receiver operating characteristic curve, *PPV* positive predictive value, *NPV* negative predictive value, *LR* logistic regression, *RF* random forest, *LGBM* light gradient boosting model, *MLP* multilayer perceptron, CNN convolutional neural network.

^a^The model using only clinical features.

^b^The model using only images.

^c^The model using both clinical features and images.

**Figure 2:**
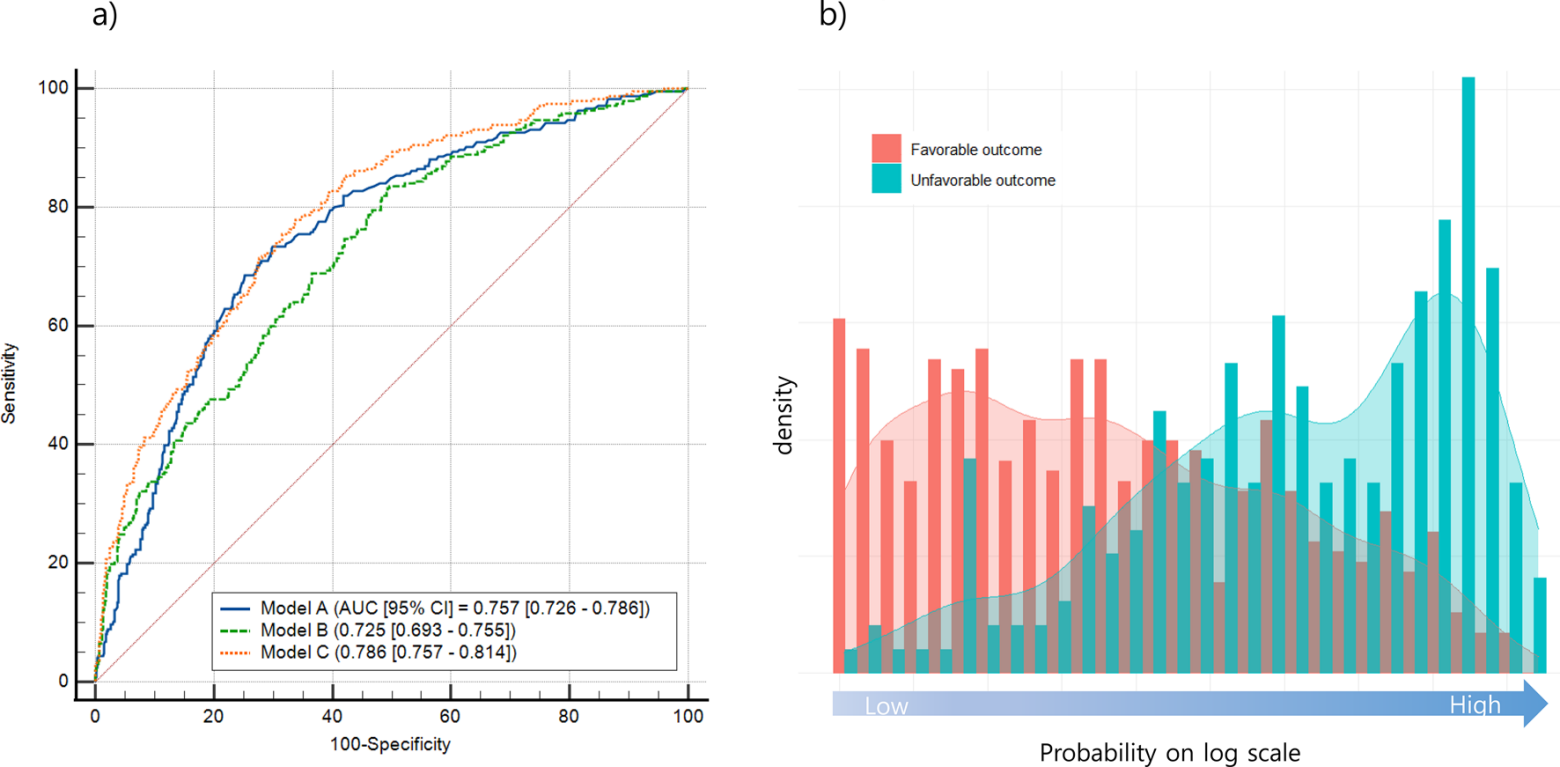
Receiver operating characteristic curves (left) and probability distribution (right) of binomial prediction models for predicting 3-month functional outcome in test set. Probability distribution from Model C were illustrated. For the x-axis, log scale was applied.

In the test set, the AUCs of Models A, B, and C were 0.757 (95% CI 0.726–0.789), 0.725 (95% CI 0.693–0.755), and 0.786 (95% CI 0.757–0.814), respectively. Model C exhibited a significantly higher AUC compared to Models A and B (difference in the AUCs between Models A and C: 0.032, 95% CI 0.009–0.051, P < 0.0058; Model B and C: 0.062, 95% CI 0.033–0.091, P < 0.0001) (Fig. 2). The Brier scores of Models A, B, and C were 0.175, 0.178, and 0.164, respectively.

Model calibration was performed to assess the likelihood that a given new observation belongs to each of the known classes. The calibration slopes showed minimal difference between the predicted and observed probability of unfavorable outcomes, indicating a good model fit (Fig. S10).

**Figure 3:**
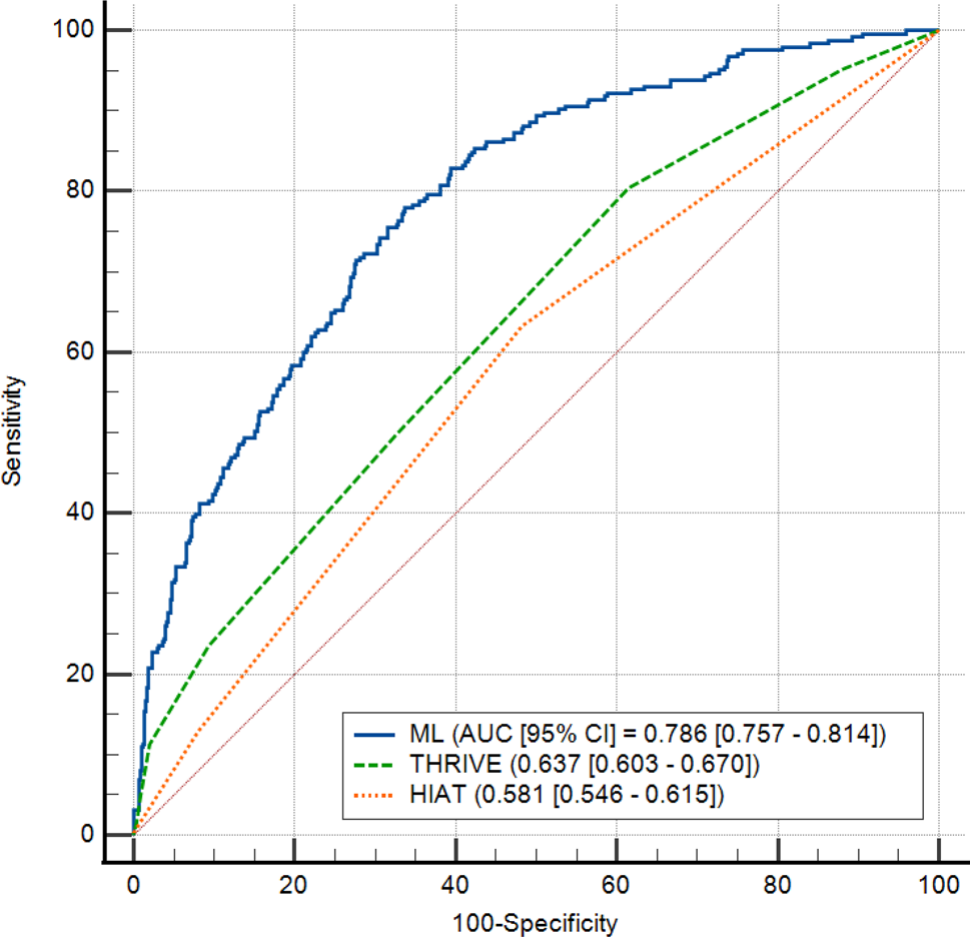
Receiver operating characteristic curves of the previous models and the integrated model predicting 3-month functional outcome in the test set.

In the test set, Model C (ML) showed a significantly higher AUC than that of the traditional models (HIAT and THRIVE) (difference in the AUCs between ML and HIAT in the test set: 0.206, 95% CI 0.159–0.252, P < 0.0001; ML and THRIVE: 0.149, 95% CI 0.105–0.193, P < 0.0001) (Fig. 3).

## Discussion

In this study, we proposed an ML system that automatically segments infarct lesions and classifies 3-month functional outcomes by combining a CNN model using imaging data with a clinical model utilizing optimal clinical features. The system was developed with a multi-center training set of 3,332 patients and its performance was validated using a test set of 822 patients. The segmentation model displayed good performance in infarct core lesion segmentation. The results demonstrated that the integrated model was superior to both the clinical and imaging models. The integrated ML model outperformed traditional risk-scoring models, and the classification results of the imaging model and Grad-CAM suggested that the imaging model accurately detected infarction lesions and might have learned the eloquent brain area.

Concerns regarding overfitting and the need for input convenience in clinical practice prompted us to select features for use as input variables. As a result, Age, NIHSS, and END were identified as the most important features (Fig. S7). These are well-known risk factors for stroke functional outcomes and have been consistently reported in previous studies^8,9,28–30^. To not only assess the strength of these selected variables' contributions to the outcome but also to understand their direction of influence, we examined the SHAP values and found that they reflected the anticipated clinical direction of influence (Fig. S8). Furthermore, these features exhibited the same importance and directionality in the generalized linear model. Using logistic regression, the odds ratios (OR) for an unfavorable outcome from Age, NIHSS, and END were 1.04 (95% CI 1.03–1.04), 1.24 (1.21–1.27), and 6.90 (5.32–8.95) respectively. While the effect sizes of Initial NIHSS and Age might seem negligible at first glance, considering the increase in log(odds) with every unit increase of these variables reveals a profound impact.

In our study, Model S (segmentation model) effectively segmented infarct core lesions. The model can visualize the infarction lesion and calculate the core volume. While the predicted mask from the segmentation model was utilized as an input for the classification model, its contribution to improving prediction performance appeared limited. However, the segmentation model still holds value for several reasons.

Firstly, by offering visual information on cerebral lesions, clinicians were able to gain essential insights into the presence and location of cerebral infarctions. This information can be particularly valuable for medical professionals who are not well-acquainted with cerebral infarction imaging.

Secondly, the segmentation model can serve a role in verifying model reliability. When faced with input images of suboptimal quality or those deemed inappropriate, clinicians have the opportunity to detect such issues early through the segmentation model. Consequently, this gives them a chance to determine the trustworthiness of the classification model's results.

Lastly, considering recent clinical trials (RESCUE-Japan LIMIT, SELECT 2, ANGEL-ASPECT)^31–33^, the volume of the cerebral infarction is deemed one of the critical determinants for recanalization therapy. The volume information of the infarct core provided by our segmentation model could be of immense help in such clinical decision-making processes.

One of the interests of our research was to determine if the brain imaging model was capturing not just the volume of the cerebral infarct but also its positioning within functionally eloquent brain areas. The term "brain eloquence" refers to the functional importance of specific brain regions, indicating their central role in neural operations. For instance, damage to crucial areas such as the primary motor cortex or language-associated regions can lead to severe clinical symptoms, whereas an infarct of similar size in other regions might not be as consequential.

**Figure 4:**
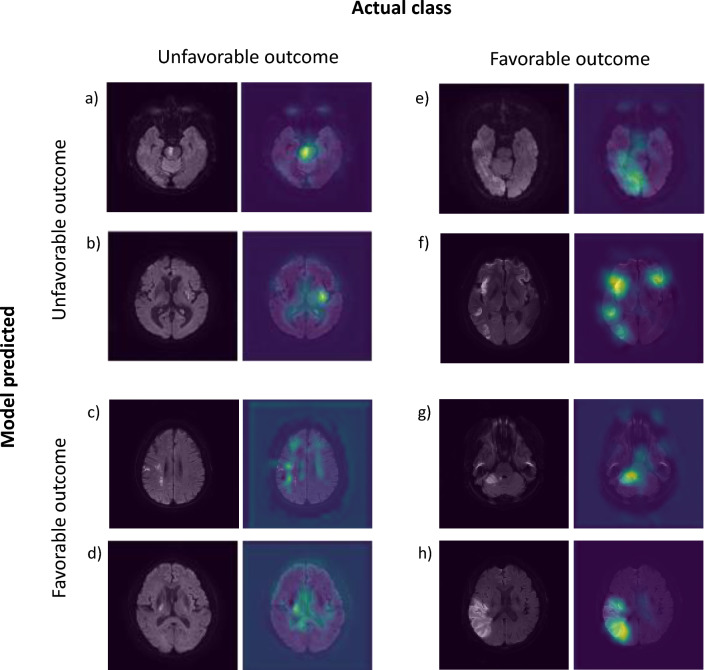
Visualization using gradient-weighted class activation mapping (Grad-CAM).

To illustrate from our study results, Model B categorized cases 'a' and 'b' as having unfavorable outcomes (as shown in Fig. 4). However, for what are considered less functionally eloquent lesions, cases 'g' and 'h' in Fig. 4 were classified with favorable outcomes, despite having a larger volume than 'a' and 'b'. These findings hint that our imaging model might be taking into account not just the volume but possibly also the location and its functional eloquence.

This consideration of functional eloquence might be especially decisive for smaller infarcts. Referring to Fig. S11, smaller infarcts, on average, didn't seem to predict clinical outcomes based on volume. However, for medium to large infarcts, there appeared to be a linear trend where an increase in volume correlated with a higher likelihood of unfavorable outcomes. This pattern has been corroborated in previous studies, which proposed that medium to large infarcts had a stronger correlation with adverse outcomes, while smaller infarcts did not^34^. The more pronounced influence of volume over location in large infarcts might be attributable to a floor effect associated with outcomes.

In summary, our imaging model suggests that for smaller infarcts, the functional eloquence of the location seems pivotal, whereas, for larger infarcts, volume takes precedence. These insights provide a comprehensive interpretation reflecting the intricate decision criteria of the model.

In this study, the utilization of Grad-CAM as a CNN-specific attribution method carries two primary implications. Firstly, it provides a foundational level of trust in the model's prediction outcomes. If the heatmap focuses on areas outside of the brain, it might indicate that the CNN model has not learned the correct patterns from the training data or might have overfitted to the training set. Without tools like Grad-CAM, relying solely on the model's probabilistic outcomes might fail to recognize these issues. Secondly, the heatmap visually demonstrates which part of the image most influences the classification decision, thereby offering insights into the model's classification criteria.

However, it's vital to note that Grad-CAM does not provide a definitive rationale for predictions. Even if the model focuses on a specific region, that region might not necessarily be the primary basis for classification. Activation intensity might not always align with the significance in class decisions, and sometimes, less activated areas might have a more significant impact on the decision. Furthermore, Grad-CAM inherently does not provide quantitative information. Its main role serves as a starting point to infer the model's classification rationale, with the interpretation largely left to developers and clinicians.

In this context, our heatmap seems to suggest two possible interpretations. Firstly, while it is challenging to explain definitively, when observing that the heatmap increases uniformly across the entire stroke rather than just a part of it, it suggests the possibility that the model has captured the volume information of the lesion. Previous studies indicating a relationship between the volume of the stroke and outcomes 3 months later support the benefits gained from capturing this volume information. Secondly, the inclusion of non-contributing regions like the ventricles, eyeballs, and air-tissue boundaries might hint at the possibility that these areas were used as landmarks to recognize the relative position of the lesion. Since our study did not spatially align brain images to a specific atlas, using such landmarks to determine the relative position of the lesion might aid in discerning whether that area is related to essential brain regions. Yet, such interpretations remain speculative, echoing the sentiments shared across most CNN-based studies where the onus of deciphering the model's workings is entrusted to developers and clinicians.

This study had several limitations. First, due to the retrospective nature of the investigation, the performance results may be insufficient to determine the robustness of the ML model on clinical utility. Well-designed prospective cohort studies are required to provide clear evidence for clinical use. Second, the proposed ML model uses only DWI obtained at one time point during admission. Using image data from additional timepoints or other imaging modalities may be associated with additional uncaptured performance. The use of further advanced imaging or bio-signal data, such as vital signs, Holter ECG, or EEG, as additional features may aid ML models in learning deeper pathophysiologic mechanisms of ischemic strokes. Third, this study's lack of external validation raises concerns about its generalizability. However, this study utilized a large dataset from three stroke centers, and the unseen data only for the test was separately validated.

In conclusion, this study proposed an ML system that combines a CNN model using imaging data with a clinical model utilizing optimal clinical features to automatically segment infarct lesions and classify 3-month functional outcomes in stroke patients. The integrated model demonstrated superior performance compared to the clinical model, imaging model, and traditional risk-scoring models. The study also explored the use of Grad-CAM to visualize the prediction basis for the regions of the brain the CNN model used for classification.

### Supplementary Information

Supplementary Information.

## Data Availability

The data analyzed in this study is subject to the following licenses/restrictions: access to the data from the National Information Society Agency (https://aihub.or.kr/) has been granted, but the data is only available for review by domestic residents who have received approval. Requests to access these datasets should be directed to corresponding author.
