# Supplementary material for: Combining clinical and imaging data for predicting functional outcomes after acute ischemic stroke: an automated machine learning approach
Source: Sci Rep. 2023 Oct 7;13:16926. doi: 10.1038/s41598-023-44201-8 (PMC10560215; doi:10.1038/s41598-023-44201-8)
Supplement: Supplementary file 1 — Supplementary Information. [file 41598_2023_44201_MOESM1_ESM.pdf]

Supplemental Materials

Table S1. Common clinical variables

**Demographics**	Smoking
Age	Atrial fibrillation
Gender	History of stroke
**Baseline variables**	History of cancer
BMI – kg/㎡	History of coronary heart disease
Systolic BP – mmHg	History of peripheral artery disease
Diastolic BP – mmHg	**Laboratory examinations**
**Stroke assessment and treatment**	WBC – 10^9/L
Pre-stroke mRS	Hemoglobins – g/dl
NIHSS score at baseline	Hematocrit – ratio
TOAST	PT – INR
END*	Serum glucose – mg/dl
Recanalization therapy†	BUN – mg/dl
**Stroke risk factors**	Creatinine – mg/dl
Stroke onset to admission delay	HDL – mg/dl
Hypertension	LDL – mg/dl
Diabetes mellitus	Total cholesterol – mg/dl
Dyslipidemia	Triglycerides – mg/dl

BMI, body mass index; mRS, modified Rankin Scale; NIHSS, National Institutes of Health Stroke Scale; TOAST, Trial of Org 10172 in Acute Stroke Treatment[1]; END, Early neurologic deterioration;

* END was defined as an increase in the NIHSS score of 1 or more from admission for acute cerebral infarction until discharge.

† Whether acute recanalization treatment such as intravenous tissue plasminogen activator or intra-arterial thrombectomy was performed.

Figure S1. De-identification method

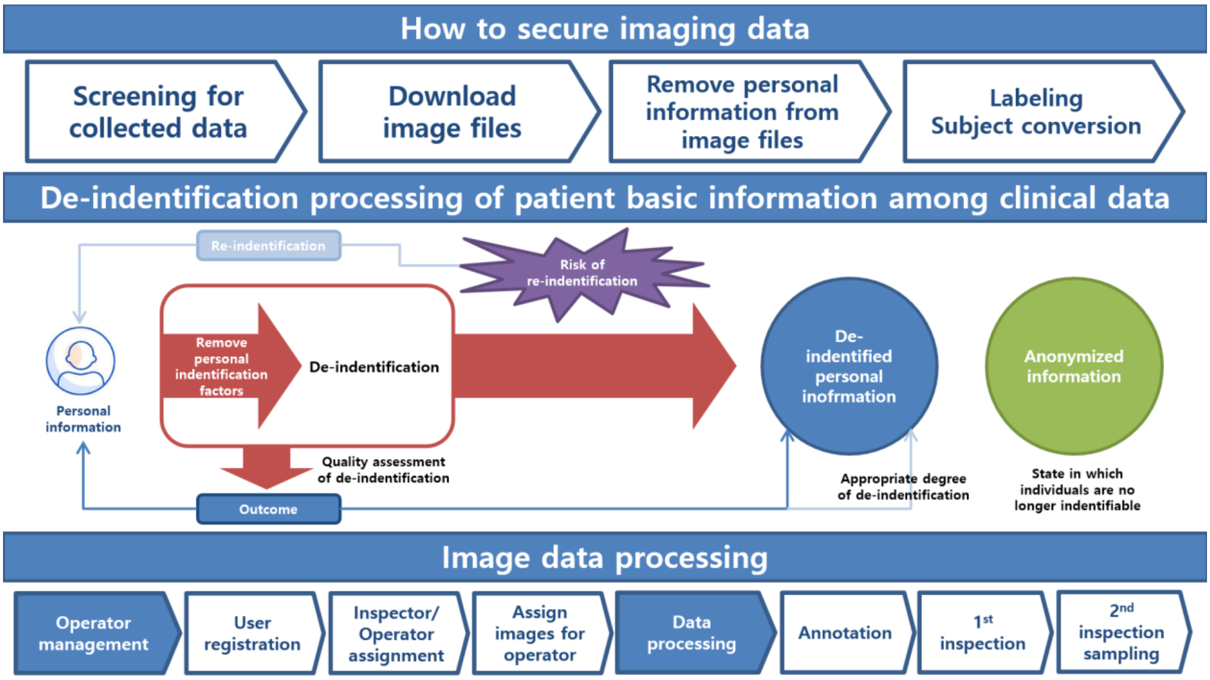

Method S1. Clinical data preparation and feature selection

Outlier detection and imputation for missing values below 10% were performed on the clinical data of the entire included derivation set. Two experienced clinicians (P.K.M and L.J.W) examined the distributions of the 31 common variable values, identifying any clinically implausible levels. Outlier candidates were replaced with NAs based on clinicians' consensus. Multivariate Imputation by Chained Equations (MICE) was conducted on both the NA-replaced outliers and the original data's missing values. [2]

To ensure the proposed model's usability in clinical practice and minimize the risk of overfitting, we carried out a feature selection process to identify the minimal input features. Using univariable logistic regression, random forest feature importance, permutation importance, and Shapley Additive Explanations (SHAP) methods,[3] we determined features with significant influence on the outcome among all 31 common variables.

Random forest feature importance measures the importance of each variable based on the average decrease in impurity across decision tree nodes within the model. Higher importance values indicate a larger impact on the model's prediction performance. Permutation Importance assesses feature importance in a model-agnostic manner, involving random shuffling of a single feature's values and measuring the change in the model's performance. A larger performance drop indicates higher importance for that feature. The SHAP method identifies important features by calculating their contribution to model predictions. High absolute SHAP values indicate more influential features suitable for model training.

Based on the results of the methods above, several covariates for building the prediction model were determined through the researchers' review of the identified features. By considering the importance and influence of each feature on the model's predictive performance, the researchers were able to select a set of variables that would yield a more accurate and reliable prediction model. This streamlined model allows for efficient implementation in clinical practice while minimizing the risk of overfitting and maintaining its predictive power.

Method S2. MR image preparation and acquisition protocol

Data preparation

All lesions in the diffusion-weighted images were manually segmented by experienced clinicians. During this process, images with severe distortion due to susceptibility artifacts that made segmentation impossible, as well as lesions not due to the mechanism of ischemic stroke, were excluded from the dataset.

Acquisition protocol

- Hospital A

1.5 T MRI scanner (Signa, GE Medical System)

DWI and ADC was performed using echo-planar pulse sequences

(TR/TE = 9000/81ms, FA = 90°, slice thickness = 4.0 mm, acquisition matrix = 128 × 128, FOV = 260 × 260 mm2, and bvalue = 1 ,000 s/mm2, ADC ≤620×10−6 mm2/s).

- Hospital B

1.5 T MRI scanner (Avanto, Siemens)

DWI and ADC was performed using echo-planar pulse sequences

(TR/TE = 5700/76ms, FA = 90°, slice thickness = 3.0 mm, acquisition matrix = 162 × 146, FOV = 240 × 240 mm2, and bvalue = 1,000 s/mm2, ADC ≤620×10−6 mm2/s).

- Hospital C

3.0 T MRI scanner (Ingenia, Phillips Medical System)

DWI and ADC was performed using echo-planar pulse sequences

(TR/TE = 4310/99ms, FA = 90°, slice thickness = 3.0 mm, acquisition matrix = 128 × 126, FOV = 220 × 220 mm2, and bvalue = 1,000 s/mm2, ADC ≤620×10−6 mm2/s).

Figure S2. Image pre-processing

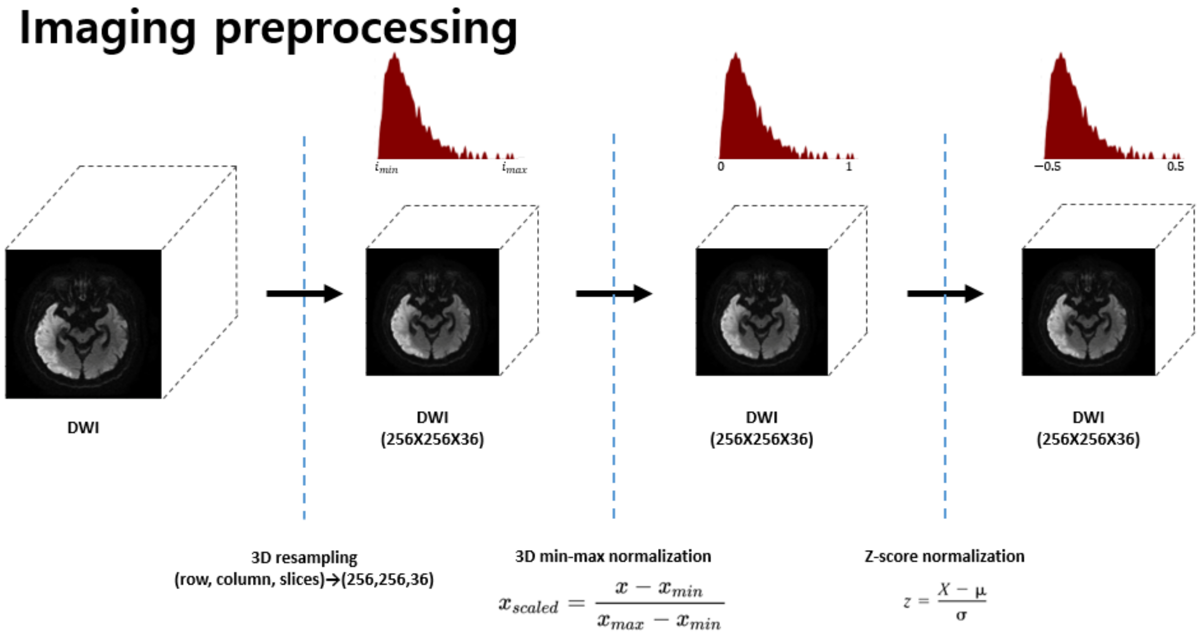

Figure S3. K-fold cross validation

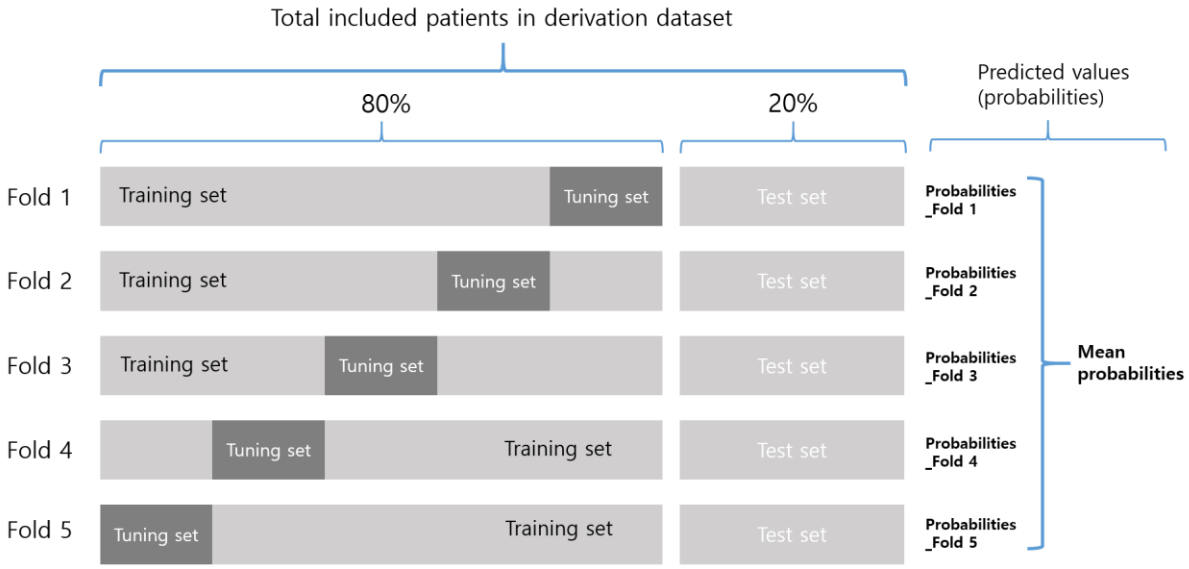

We randomly allocated 20% of the dataset as a test set, exclusively for evaluation. The remaining 80% served as a training set for hyperparameter determination and training, employing 5-fold cross-validation. Consequently, we built five models, each assessed using the same test set. We used the mean predicted values (probabilities) inferred from each model for the outcome label to validate performance.

Figure S4. Flow diagram of machine learning model development and evaluation

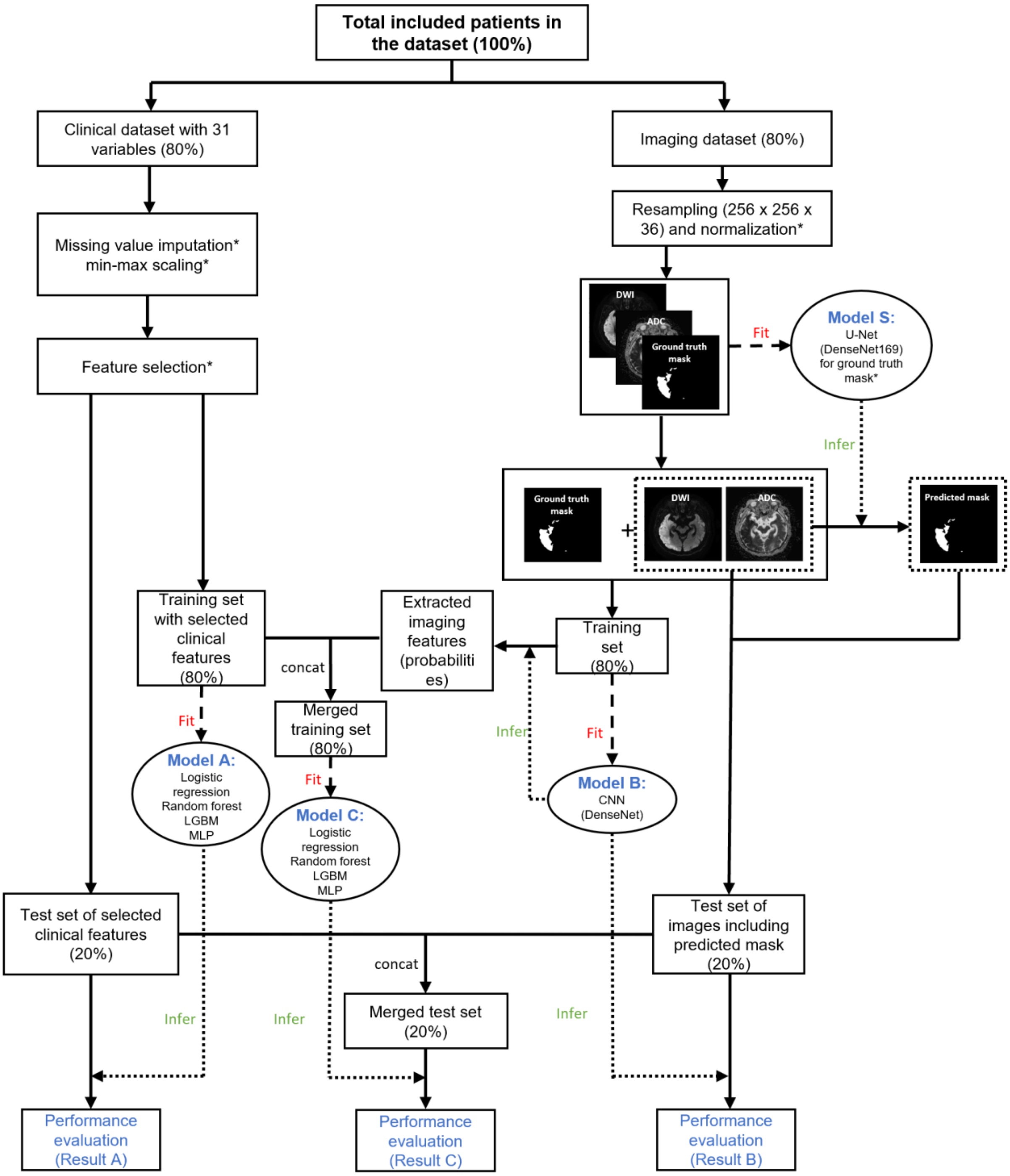

Model S : Lesion segmentation model using U-Net with a ResNet152 backbone.

Model A : the model using only clinical features.

Model B : the model using only images (DWI, ADC, infarction lesion mask).

Model C: the model using both class probability for unfavorable outcomes inferred from Model B and clinical features.

DWI, diffusion weighted image; ADC, Apparent diffusion coefficient; CNN, Convolutional Neural Networks; LGBM, Light Gradient Boosting Model; MLP, multilayer perceptron.

Figure S5. Patient flowchart

a)

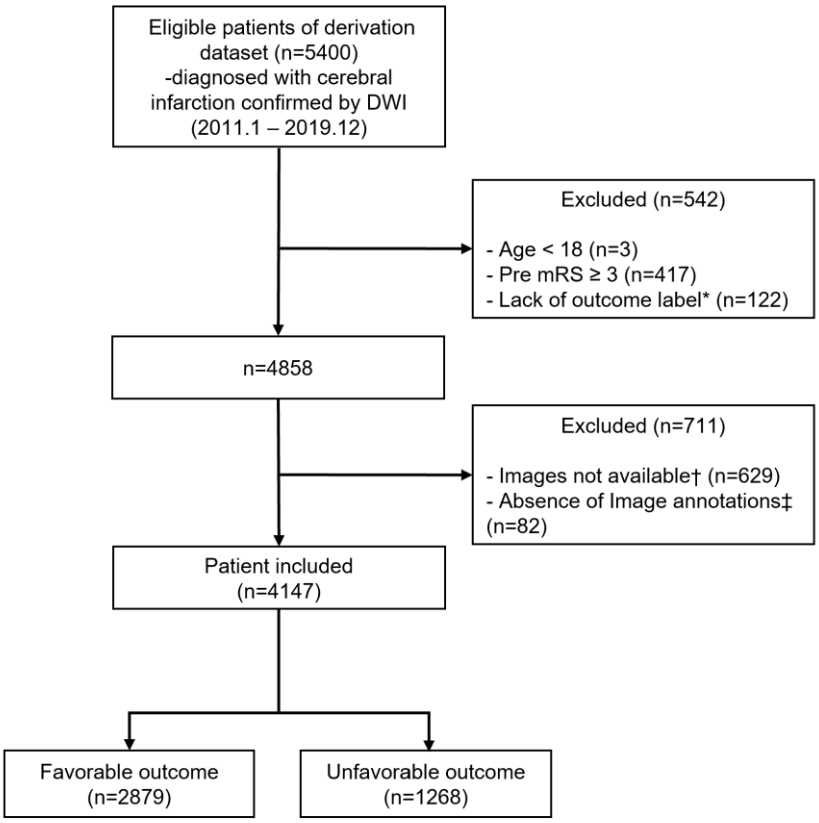

* Outcome label indicates 3 month Modified Rankin Score.

† Cases in which neither the DICOM file, the diffusion weighted image, the apparent diffusion coefficient image, or ground truth image labels (manually annotated infarcted lesions) are provided.

‡ Cases without labels that segmented cerebral infarction lesions.

n, number of patients; DWI, diffusion weighted imaging; Pre mRS, Pre-Stroke Modified Rankin Score, Favorable outcome, month Modified Rankin Score ≤ 2; Unfavorable outcome, 3 month Modified Rankin Score > 2; DICOM, Digital Imaging and Communications in Medicine.

Figure S6. Missing value patterns according to the clinical variables.

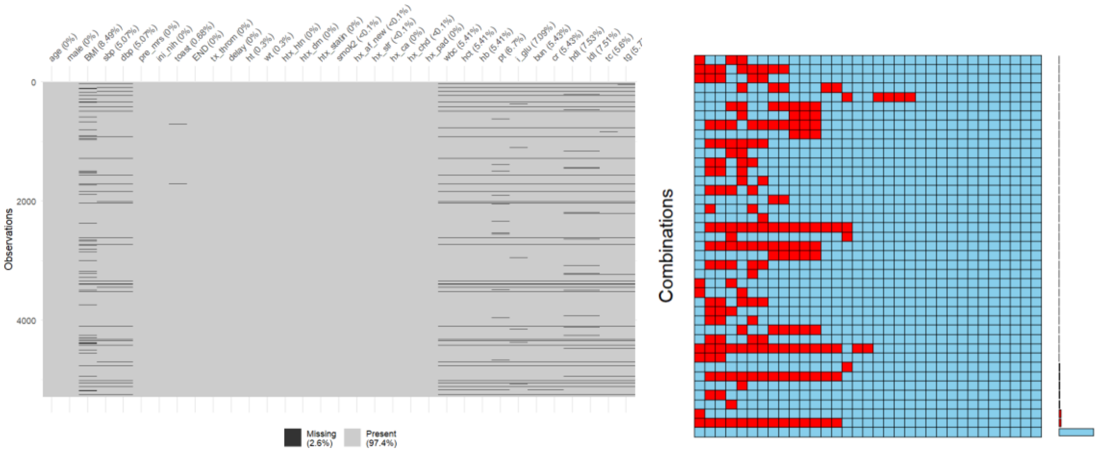

Table S2 Baseline characteristics

Variables	Favorable outcome (N=2879)	Unfavorable outcome (N=1268)	P-value
Male – no. (%)	1765 (61.3)	686 (54.1)	<0.001
Age – year	66.5 ± 12.6	71.7 ± 11.7	<0.001
BMI – kg/㎡	24.0 ± 3.3	30.0 ± 77.8	<0.001
Prestroke mRS			0.010
0	2276 (79.1)	974 (76.8)	
1	426 (14.8)	183 (14.4)	
2	177 (6.1)	111 (8.8)	
NIHSS score at baseline (IQR)	2 (1-4)	4 (2-8)	<0.001
TOAST – no. (%)			<0.001
LAA	915 (31.8)	449 (35.4)	
SVO	608 (21.1)	217 (17.1)	
CE	428 (14.9)	251 (19.8)	
Other determined	60 (2.1)	25 (2.0)	
Undetermined	868 (30.1)	326 (25.7)	
Onset to admission delay – no. (%)			0.127
< 3 hr	949 (33.0)	411 (32.4)	
3-6 hr	397 (13.8)	210 (16.6)	
6-12 hr	372 (12.9)	166 (13.1)	
12-24 hr	331 (11.5)	164 (12.9)	
24-36 hr	247 (8.6)	98 (7.7)	

36-48 hr	103 (3.6)	41 (3.2)	
48 hr -1 week	423 (14.7)	155 (12.2)	
>1week	57 (2.0)	23 (1.8)	
END – no. (%)	129 (4.5)	255 (20.1)	<0.001
Acute treatment – no. (%)			<0.001
No treatment	2539 (88.2)	1051 (82.9)	
IV tPA	210 (7.3)	122 (9.6)	
IA Thrombectomy	49 (1.7)	52 (4.1)	
Both	81 (2.8)	43 (3.4)	
Hypertension – no. (%)	1202 (41.8)	615 (48.5)	<0.001
Diabetes mellitus – no. (%)	577 (20.0)	305 (24.1)	0.004
Dyslipidemia – no. (%)	306 (10.6)	122 (9.6)	0.354
Atrial fibrillation – no. (%)	423 (14.7)	228 (18.0)	0.007
Smoking – no. (%)			<0.001
Never	1987 (69.0)	955 (75.3)	
Current	614 (21.3)	204 (16.1)	
Ex-smoker (>5yr)	104 (3.6)	57 (4.5)	
Ex-smoker (<5yr)	174 (6.0)	52 (4.1)	
Previous history – no. (%)			
Stroke	384 (13.3)	254 (20.0)	<0.001
Cancer	36 (1.3)	19 (1.5)	0.620
Coronary heart disease	159 (5.5)	83 (6.5)	0.221
Peripheral artery disease	11 (0.4)	13 (1.0)	0.022
WBC – 10^9/L	7.9 ± 2.7	8.4 ± 3.1	<0.001
Hemoglobins – g/dl	13.9 ± 1.8	13.5 ± 2.0	<0.001
Hematocrit – ratio	40.8 ± 5.1	39.6 ± 5.8	<0.001
PT – INR	1.0 ± 0.2	1.1 ± 0.2	<0.001
Serum glucose – mg/dl	137.3 ±53.9	142.2 ± 58.9	0.011
BUN – mg/dl	16.1 ± 6.1	17.5 ± 7.1	<0.001
Creatinine – mg/dl	0.9 ± 0.5	0.9 ± 0.6	0.012
HDL – mg/dl	45.2 ±15.8	44.8 ±12.3	0.314
LDL – mg/dl	111.0 ±38.8	109.1 ±39.9	0.141
Total cholesterol – mg/dl	176.5 ±44.6	173.8 ±46.4	0.074
Triglycerides – mg/dl	131.0 ±82.0	120.3 ±83.5	<0.001
Systolic BP – mmHg	142.6 ± 26.0	143.8 ± 26.5	0.151
Diastolic BP – mmHg	84.2 ± 14.1	83.6 ± 14.9	0.263

BMI, body mass index; mRS, modified Rankin Scale; NIHSS, National Institutes of Health Stroke Scale; TOAST, Trial of Org 10172 in Acute Stroke Treatment[1]; END, Early neurologic

deterioration; tPA, tissue plasminogen activator; WBC, white blood cell; PT-INR, prothrombin time international normalized ratio; BUN, blood urea nitrogen; HDL, high-density lipoprotein; LDL, low-density lipoprotein

mRS : Scores on the modified Rankin scale range from 0 to 6, with higher scores indicating greater disability

NIHSS : Scores on the National Institutes of Health Stroke Scale (NIHSS) range from 0 to 42, with higher scores indicating greater deficit.

Figure S7. Selecting clinical features that contribute to functional outcomes using a) Random Forest feature importance, b) permutation importance, and c) absolute mean SHAP value.

a)

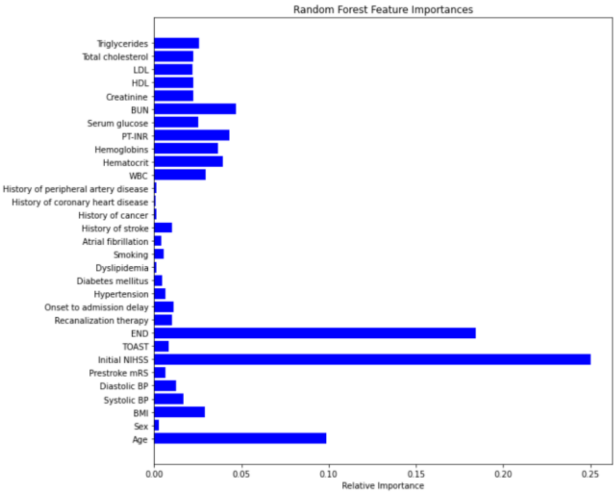

b)

Weight	Feature
0.0887 ± 0.0364	Initial NIHSS
0.0850 ± 0.0455	END
0.0225 ± 0.0064	Age
0.0136 ± 0.0088	Hemoglobins
0.0133 ± 0.0057	Hematocrit
0.0096 ± 0.0091	BUN
0.0068 ± 0.0062	Prestroke mRS
0.0058 ± 0.0024	Total cholesterol
0.0056 ± 0.0100	PT-INR
0.0049 ± 0.0066	HDL
0.0030 ± 0.0039	Creatinine
0.0029 ± 0.0069	Smoking
0.0029 ± 0.0041	Onset to admission delay
0.0024 ± 0.0024	Sex
0.0017 ± 0.0079	BMI
0.0016 ± 0.0098	WBC
0.0013 ± 0.0048	Atrial fibrillation
0.0010 ± 0.0081	Hypertension
0.0006 ± 0.0046	Recanalization therapy
0.0004 ± 0.0036	Systolic BP
... 11 more ...	

c)

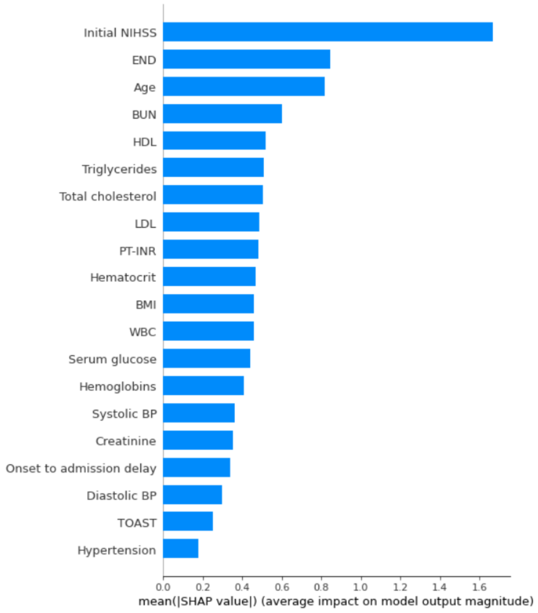

Figure S8. SHAP summary plot. This plot visualizes the contribution of each feature in explaining a model's predictions. The three most important features were initial NIHSS, END, and Age, in that order, which all positively contributed to the prediction of unfavorable outcomes as their values increased. Conversely, as the values of HDL, TG, and LDL decreased, they contributed to the prediction of unfavorable outcomes. The relationship between the distribution of variable values and SHAP values was less heterogeneous in initial NIHSS, END, Age, Onset to admission delay, and Hypertension compared to other features.

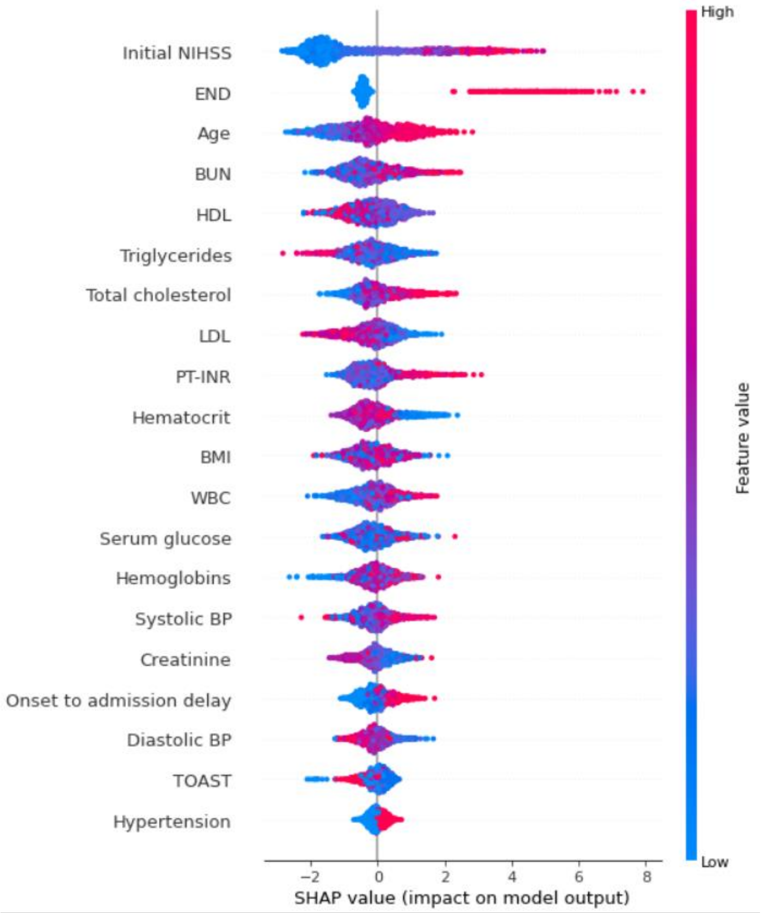

NIHSS, National Institutes of Health Stroke Scale; END, Early neurologic deterioration; BUN, blood urea nitrogen; HDL, high-density lipoprotein; LDL, low-density lipoprotein; PT-INR, prothrombin time international normalized ratio; BMI, body mass index; WBC, white blood cell; TOAST, Trial of Org 10172 in Acute Stroke Treatment

* END was defined as an increase in the NIHSS score of 1 or more from admission for acute cerebral infarction until discharge.

Table S3. Tuned hyperparameter for the machine learning models.

Algorithm	Parameters
	**Model A**
LR	- sklearn default
RF	- n_estimators=50, max_depth=2
LGBM	- n_estimators=100, learning_rate=1e-2, max_depth=2, num_leaves=5, subsample=0.7
MLP	- hidden_layer_1=100, hidden_layer_2=50, Leaky ReLU, BatchNorm, He initialization, dropout=0.2<br>- optimizer=AdamW(learning_rate=1e-4, betas=(0.9, 0.999), eps=1e-08, weight_decay=1e-5)<br>- batch_size=64<br>- loss=CrossEntropyLoss
	**Model B**
CNN	3D DenseNet 169<br>- optimizer=Adam(learning_rate=1e-4)<br>- batch_size=16<br>- loss=CrossEntropyLoss
	**Model C**
LR	- sklearn default
RF	- n_estimators=120, max_depth=4
LGBM	- n_estimators=200, learning_rate=1e-2, max_depth=4, num_leaves=5, subsample=0.7
MLP	- hidden_layer_1=200, hidden_layer_2=100, Leaky ReLU, BatchNorm, He initialization, dropout=0.2<br>- optimizer=AdamW(learning_rate=1e-4, betas=(0.9, 0.999), eps=1e-08, weight_decay=1e-5)<br>- batch_size=64<br>- loss=CrossEntropyLoss

LR, logistic regression; RF, random forest; LGBM, light gradient boosting model; MLP, multilayer perceptron; CNN, Convolutional Neural Networks.

Table S4. Computational time (inference time) for the machine learning models

The integrated model (Model C) was run with the original diffusion weighted image, initial NIHSS, age, and END, and the length of time it took to get the outcomes (predicted probability, predicted mask, and Grad-CAM) was reported.

Model	LR	RF	LGBM	MLP
Inference time [sec]	21.25	20.85	21.21	20.61

Python version : 3.7.15

Pytorch version : 1.12.1

CPU : Intel(R) Xeon(R) CPU @ 2.20GHz 12cores

GPU : A100-SXM4-40GB

Table S5. Area under the receiver operating characteristic curves according to fold and ML models

	**Model A**				**Model B**	**Model C**			
	LR	RF	LGBM	MLP	CNN	LR	RF	LGBM	MLP
Fold 1	0.764	0.754	0.753	0.763	0.728	0.780	0.782	0.782	0.782
Fold 2	0.764	0.755	0.751	0.765	0.716	0.774	0.776	0.773	0.781
Fold 3	0.765	0.758	0.752	0.764	0.719	0.778	0.777	0.779	0.779
Fold 4	0.764	0.754	0.754	0.765	0.727	0.782	0.785	0.781	0.784
Fold 5	0.765	0.754	0.750	0.764	0.725	0.775	0.775	0.774	0.775

Figure S9. Example results of the segmentation model

a)

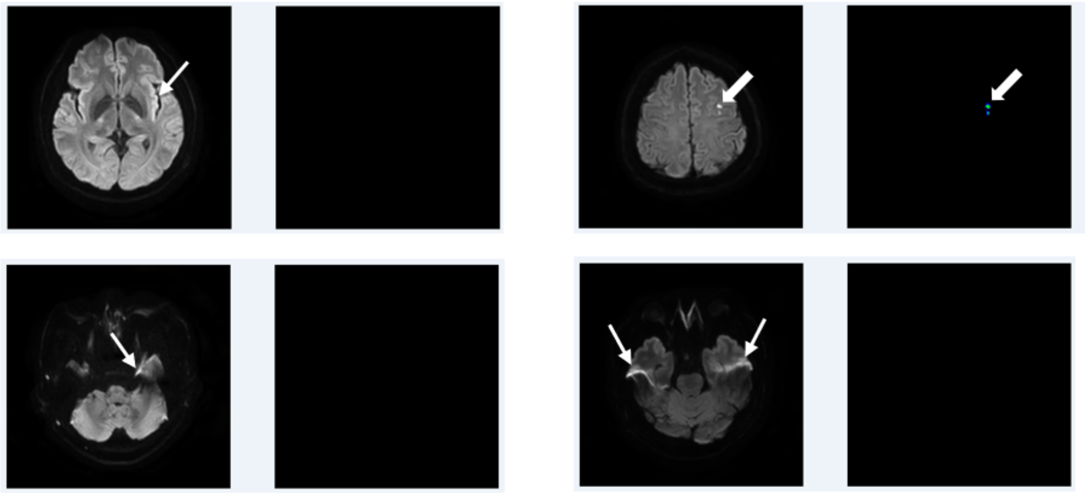

b)

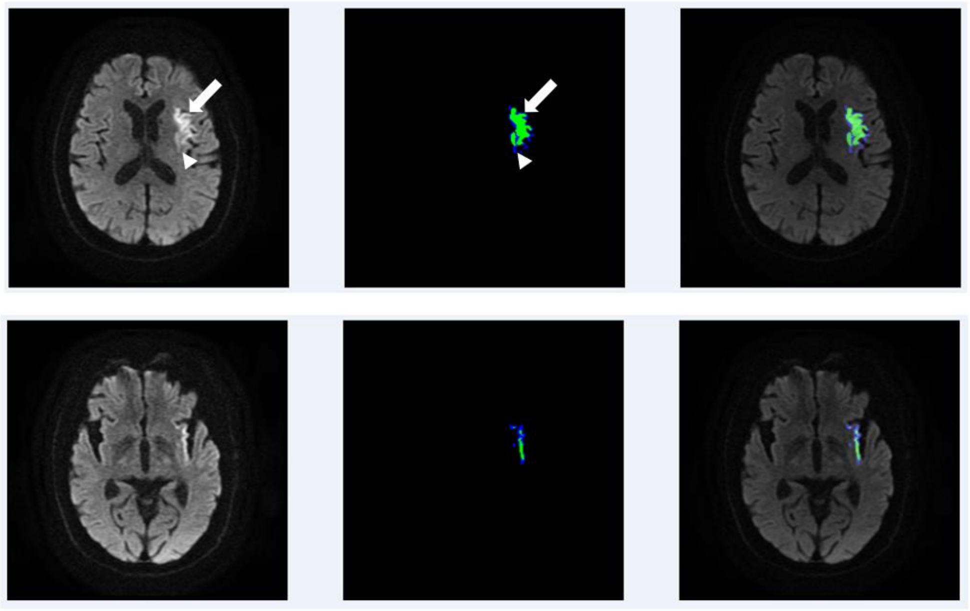

c)

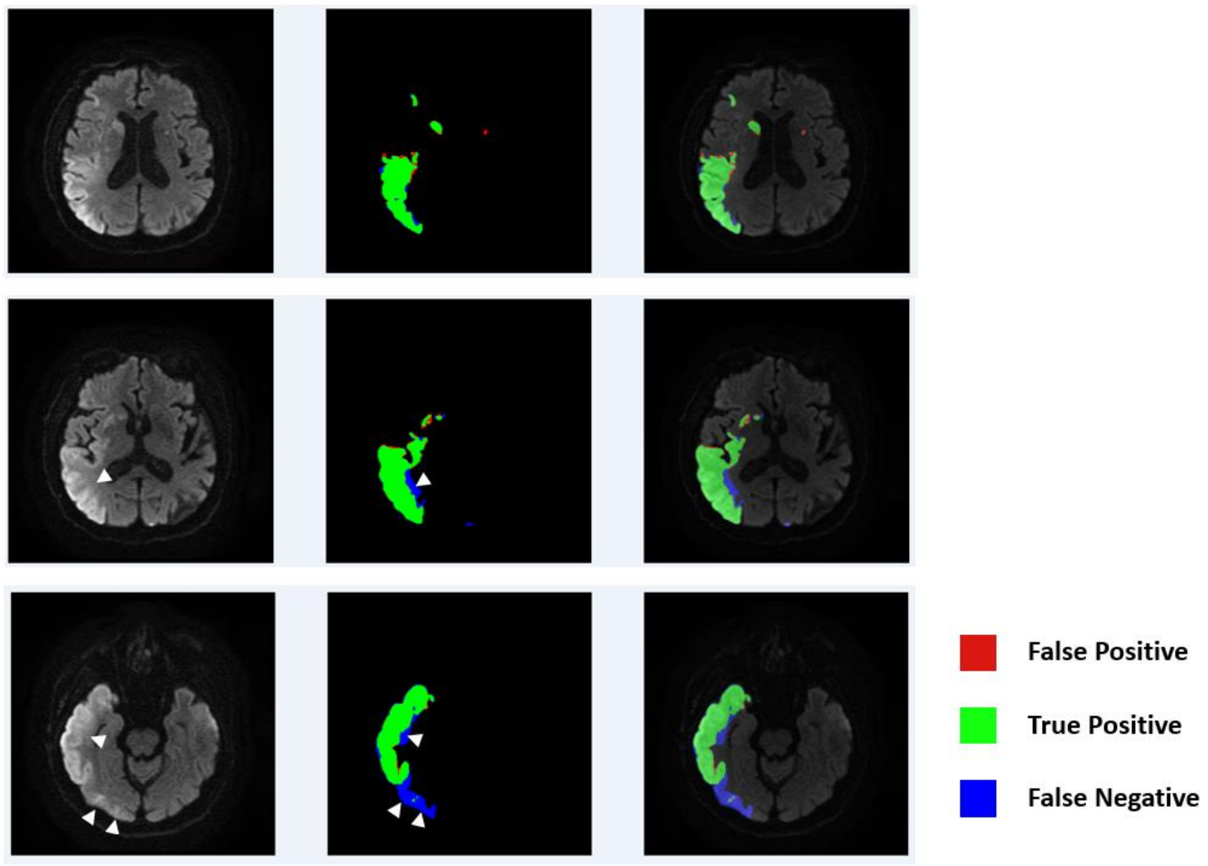

a) Small dot-like lesions (large white arrows) were accurately predicted as lesions. Model S also effectively excluded artifacts that could be mistaken for diffusion restriction, such as high signal intensities surrounding the insular cortices or pneumatized sphenoid sinus and mastoid air cells (white arrows), with only the infarct core being segmented as lesions. b, c) Lesions with distinct diffusion restriction boundaries show good concordance with the ground truth mask (large white arrow). However, lesions with ambiguous boundaries exhibit inconsistent results (arrowheads). Inconsistent labeling by humans (ground truth mask segmentation) may also contribute to these inconsistent results.

Figure S10. Calibration plots of Model A, B, and C in test set for prediction of likelihood of unfavorable outcome.

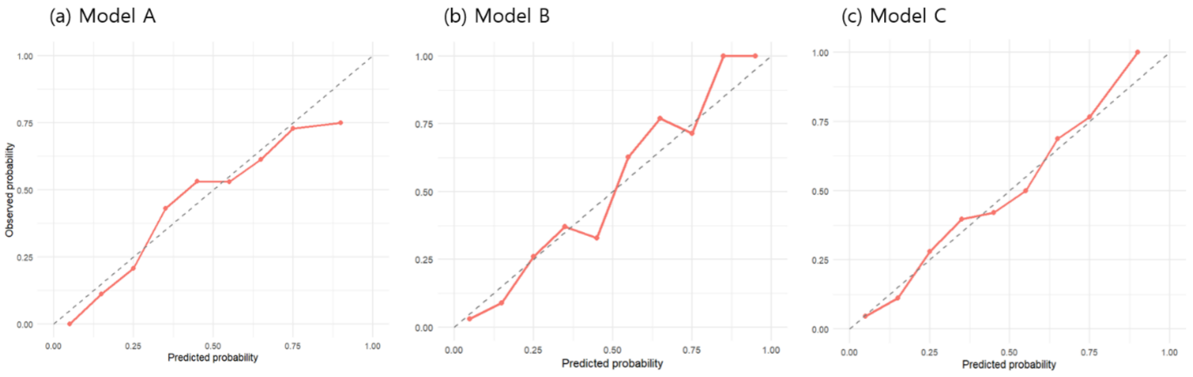

Figure S11. Probabilities of unfavorable outcome according to volumes on log scale in external validation set.

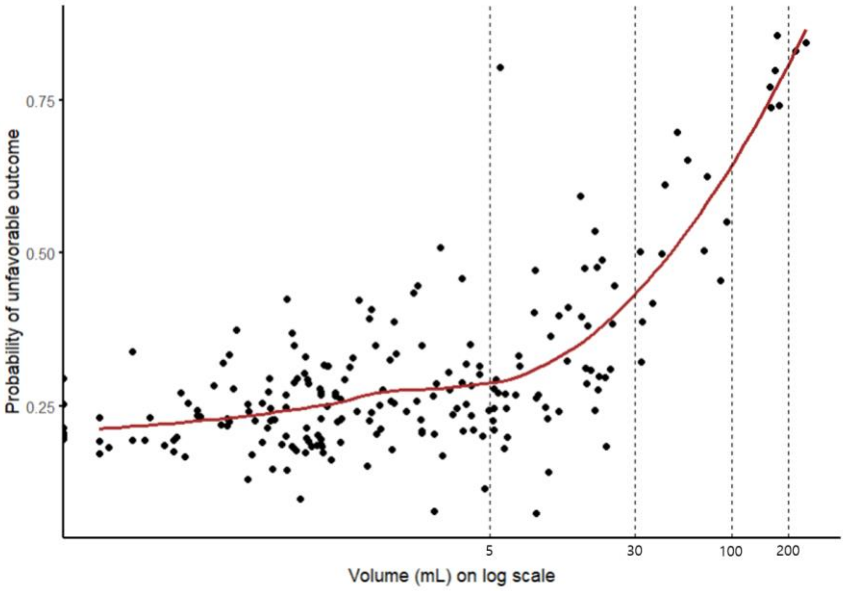

The line that best fits the results is plotted using the LOESS function. (brown line)

Reference

1. Adams Jr HP, Bendixen BH, Kappelle LJ, et al. Classification of subtype of acute ischemic stroke. Definitions for use in a multicenter clinical trial. TOAST. Trial of Org 10172 in Acute Stroke Treatment. stroke 1993;24:35-41.
2. Van Buuren S, Groothuis-Oudshoorn K. mice: Multivariate imputation by chained equations in R. Journal of statistical software 2011;45:1-67.
3. Lundberg SM, Erion G, Chen H, et al. From local explanations to global understanding with explainable AI for trees. Nature machine intelligence 2020;2:56-67.
